# Robotic-assisted versus conventional hip arthroplasty: a comparative analysis of perioperative blood management and early outcomes

**DOI:** 10.1051/sicotj/2024055

**Published:** 2024-12-23

**Authors:** Adarsh Annapareddy, Tarun Jayakumar, Manideep Reddy, Praharsha Mulpur, Vijay Kumar Reddy Gurram, Vemaganti Badri Narayana Prasad, A. V. Gurava Reddy

**Affiliations:** Sunshine Bone and Joint Institute, KIMS-Sunshine Hospitals Hyderabad India

**Keywords:** Total hip arthroplasty, Robotic-assisted surgery, Robot-assisted total hip arthroplasty, Blood loss, Blood transfusion, MAKO SmartRobotics

## Abstract

*Purpose*: This study aimed to evaluate the impact of Robotic-Assisted Total Hip Arthroplasty (RATHA) versus Conventional Total Hip Arthroplasty (CTHA) on perioperative blood loss, blood transfusion requirements, and early clinical outcomes. *Methods*: A prospective cohort study was conducted at a high-volume tertiary care center from January 2021 to January 2023. A total of 200 patients undergoing primary THA were equally divided into RATHA (using the MAKO SmartRobotics system) and CTHA cohorts. Propensity score matching adjusted for demographics and baseline characteristics, resulting in 172 matched patients. Primary outcomes included changes in perioperative hemoglobin, estimated blood loss (EBL), and transfusion rates. Secondary outcomes assessed were operative time, length of stay, and transfusion-related adverse events. *Results*: The RATHA group demonstrated significantly lower post-operative hemoglobin drops (2.49 ± 0.6 g/dL vs. 3.38 ± 1.0 g/dL; *p* < 0.001), reduced EBL on post-operative day 3 (1125.52 ± 361.2 mL vs. 1611.12 ± 501.4 mL; *p* < 0.0001), and lower transfusion rates (7.96% vs. 20.4%; p = 0.0175) compared to the CTHA group. Operative time was significantly shorter in the RATHA group (68.01 ± 8.7 minutes vs. 77.1 ± 10.5 minutes; *p* < 0.0001). All robotic cohort patients were discharged within 3 days, whereas 14% of CTHA patients required extended hospitalization. *Conclusion*: This study demonstrates that robotic-assisted total hip arthroplasty (RATHA) significantly reduces perioperative blood loss, hemoglobin drop, and blood transfusion rates compared to conventional total hip arthroplasty (CTHA). The observed decrease in operative time and hospital stay in the RATHA group further suggests that robotic assistance may enhance procedural efficiency and support faster patient recovery.

## Introduction

Primary total hip arthroplasty (THA) is a common and established surgical option for painful degenerative arthritis of the hip [1–3]. Globally, the adult hip osteoarthritis (OA) rate is about 18.70 per 100,000 [4, 5]. Despite advances in blood management strategies, elective joint replacement surgeries are thought to account for as much as 40% of packed red blood cell transfusions performed in orthopaedics [6]. THA may be associated with clinically significant blood loss, with some studies reporting more than 50% patients requiring a blood transfusion in the first three days after surgery [7]. Giving allogenic blood transfusions is the standard treatment for blood loss in THA patients, even though this approach has been demonstrated to carry a higher risk of morbidity and mortality as well as an increased risk of prosthetic joint infection (PJI) [8–10]. Development of hematomas can also pre-dispose to reoperation and an increased risk of periprosthetic joint infection (PJI) [11].

To address these concerns, multimodal blood management strategies have been developed, including preoperative hemoglobin optimization, use of regional anaesthesia, stringent postoperative transfusion criteria, and the administration of perioperative tranexamic acid (TXA) [12–14]. Robotic technology in arthroplasty has been proven to be associated with higher precision of component placement, as well as significantly lower incidence of component malalignment [15, 16]. The use of robotics in total knee arthroplasty (TKA) has also demonstrated a significant reduction in perioperative blood loss and transfusion rates when compared to conventional TKA [17]. However, further research needs to be done on how this technology affects the length of hospital stay, blood loss, and requirement for blood transfusions in patients undergoing robot-assisted total hip arthroplasty (RATHA). Theoretical causes of increased blood loss from conventional total hip arthroplasty (CTHA) include inadequate haemostasis, soft-tissue trauma, repeated instrumentation of the femoral canal and acetabulum, excessive and serial bone reaming/resection, and patient-specific causes.

The aim of this study was to compare blood loss between robotic-assisted and conventional manual THA. The primary objective of the study was to compare blood loss between RTHA and CTHA based on changes in perioperative hemoglobin, estimated amount of blood loss (EBL), relative blood loss (RBL), and postoperative blood transfusion rates. Secondary objectives consisted of comparing operative times, length of stay, and complication rates between both the cohorts. The null hypothesis of this study was that blood loss would be comparable in both cohorts.

## Materials and methods

This study was a prospective cohort study of patients undergoing primary THA by either manual/conventional methods or a CT-Based robotic system, at a high-volume tertiary care institution between January 2021 and January 2023. The choice between RATHA and CTHA was made by patient preference and self-selection, following a comprehensive discussion of both techniques by a designated patient-counselor. Two hundred consecutive cases (100 patients in each cohort) were included, with RATHA performed using the MAKO SmartRobotics technology by Stryker (Kalamazoo, MI). To minimize confounding, a post-hoc 1:1 propensity score matching analysis was performed, adjusting for age, Body Mass Index (BMI), ASA grade, and preoperative baseline hemoglobin, with a final matched sample size of 172 patients (88 in each cohort).

Institutional ethical approval was granted for this study (IEC No: SIEC/2023/536), and the study was compliant with the ethical standards delineated in the Declaration of Helsinki [18].

Inclusion criteria consisted of patients undergoing uncemented primary THA for primary osteoarthritis, rheumatoid arthritis or avascular necrosis of the femoral head. Exclusion criteria consisted of THA for conditions such as post-traumatic arthritis, hip dysplasia, and femoral neck fractures, patients with history of previous hip surgeries, ongoing anticoagulant therapy, bleeding disorders, or an ASA classification of grade 4. Patients diagnosed with anaemia based on the World Health Organization (WHO) cut-offs were also excluded from the study. Patients with an abnormal coagulation profile (consisting of Prothrombin Time-International Normalized Ratio/PT-INR, Activated partial thromboplastin time, bleeding and clotting times) were excluded from the study.

### Surgical technique

All patients were operated by a single surgeon, through the posterior approach to the hip, with trans-osseous reinsertion of the short external rotators, under spinal anaesthesia. Based on our protocol, all patients undergoing THA received intravenous TXA (10 mg/Kg body weight, with a maximum of 1 g bolus) before the incision. All patients underwent uncemented THA, with the robotic cohort receiving Stryker Accolade II uncemented femoral stem and Trident acetabular shell. Patients undergoing conventional THA received either Stryker Accolade II/Trident or DePuy Corail stem, with a Pinnacle acetabular shell. Surgical drains were used in all cases, and drains were removed on the second post-operative day per institutional protocol. All patients are mobilized with a standard physiotherapy protocol on the first post-operative day.

For patients undergoing RATHA, a pre-operative CT scan was done. The Mako robotic arm-assisted system uses CT scan data to create a patient-specific pre-operative planning for proper component size selection, accurate intra-operative stem and cup positioning, limb length and offset restoration assessment. All RATHA cases were done using an express workflow. With the 4.0 version software, surgeons can assess bone or component impingement by virtual range of motion simulation. Additional preparatory steps involved the insertion of pelvic-array bone pins into the iliac crest, through small stab-incisions of the skin which were placed prior to skin incision.

Postoperative management was consistent across both cohorts, encompassing a multifaceted approach to pain management, prophylaxis for deep vein thrombosis (DVT), antibiotic therapy, and rehabilitation. Systematic blood work was carried out to track hemoglobin levels before surgery and then serially for the first three days post-surgery.

The operative time for both patient groups was recorded from the time of skin incision until complete closure of the wound (skin-to-skin time) in minutes. Per institutional guidelines, all patients are discharged on the third post-operative day. Cases necessitating extended hospitalization were meticulously documented, to determine the cause for extended hospital stay beyond the third post-operative day.

### Outcome assessment

An external observer, not involved in the clinical management of the cohorts, was responsible for data collection, which included a comprehensive review of electronic medical records of the hospital for demographic details, BMI, ASA grading, comorbidities, and serial hemoglobin measurements. Blood hemoglobin levels were measured and documented pre-operatively, and serially thereafter on the first (24 h post-surgery), second (+48 h), and third post-operative days (+72 h).

Blood volume was first estimated using the *Nadler’s formula* [19], as follows:

$$\begin{array}{c} \mathrm{Men}:\;\mathrm{Blood}\;\mathrm{Volume}\;=\left(0.3669\times {\mathrm{Height}}^{3}\right)+\left(0.03219\times \mathrm{Weight}\right)+0.6041,\\ \mathrm{Women}:\;\mathrm{Blood}\;\mathrm{Volume}=\left(0.3561\times {\mathrm{Height}}^{3}\right)+\left(0.03308\times \mathrm{Weight}\right)+0.1833. \end{array}$$

Blood loss estimation was done using the *Gross formula* [20]. The Gross formula estimated blood loss by analyzing variations in perioperative hemoglobin levels and calculated blood volumes, employing the lowest recorded postoperative hemoglobin value for enhanced accuracy.

To account for variations in body surface area, and total blood volume, we also report blood loss in terms of relative blood loss. The result is reported as a percentage, which represents the proportion of blood lost relative to the patient’s total blood volume. Relative blood loss is calculated as follows:

$$\mathrm{Relative}\;\mathrm{Blood}\;\mathrm{Loss}\;\left(\mathrm{RBL}\right)=[\mathrm{Estimated}\;\mathrm{Blood}\;\mathrm{Loss}\;(\mathrm{EBL})/\mathrm{Total}\;\mathrm{Blood}\;\mathrm{Volume}\;(\mathrm{TBV})]\times 100.$$

### Statistical analysis

Continuous variables were represented as means with standard deviations, whereas categorical variables were expressed in terms of frequencies and proportions. Data normality was confirmed using the Shapiro-Wilk test. The evaluation of statistical significance between cohorts was assessed employing parametric tests, with *t*-tests applied for continuous variables and chi-square (*χ*^2^) tests for categorical variables. A *p*-value of less than 0.05 was predetermined as the threshold for statistical significance. Multivariate logistic regression analysis was performed to identify factors associated with blood loss, providing an adjusted assessment of the contributing variables. The statistical analysis was performed utilizing SPSS Version 24 (IBM, Armonk, NY, USA).

## Results

### Patient demographics

The mean age of the study population was 56 years (SD = 13.1), with a greater male preponderance (56.25%, *N* = 99) in both cohorts. Patients in both cohorts were matched for age, BMI, and ASA grading as summarized in Table 1.

**Table 1:** Demographics of the patient population in both cohorts.

Parameter	CTHA (*n* = 88)	RATHA (*n* = 88)	*p*-value
	Mean (SD)	Mean (SD)	
Age (years)	56.05 (13.9)	55.93 (12.3)	NS*
BMI (kg/m^2^)	24.89 (2.7)	24.83 (2.7)	NS*
Gender	*N* (%)	*N* (%)	
Men	53	46	NS^†^
Women	35	42	
ASA grade	*N* (%)	*N* (%)	
I	4	6	NS^†^
II	60	63	
III	24	19	

* Student t-test;

† Chi-square test.

### Drop in peri-operative hemoglobin

**Figure 1:**
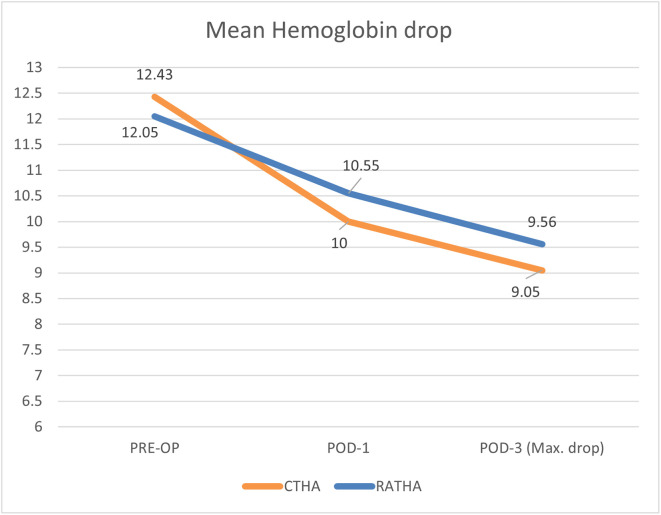
Mean hemoglobin drop during the hospital stay in both cohorts. POD-1 stands for first post-operative day.

The mean pre-operative hemoglobin (Hb) was similar in both the CTHA (12.43 ± 1.4) and RATHA (12.05 ± 1.5) cohort as shown in Table 2. However, the mean first post-operative day Hb was significantly lower in the CTHA cohort (10 ± 1.3) compared to the RATHA cohort (10.55 ± 1.4) (*p* = 0.0072). The maximal drop in hemoglobin was observed on the third postoperative day, which followed a similar trend with the CTHA (9.05 ± 1.3) having significantly lower Hb compared to RATHA (9.56 ± 1.3). The largest drop in Hb was found to be 3.38 g/dL (SD = 1) and 2.49 g/dL (SD = 0.6) in the CTHA and RATHA cohort respectively which was found to be highly significant (*p* < 0.001) (Table 2 and Figure 1).

**Table 2:** Blood parameters among CTHA and RATHA.

Parameter	CTHA (*n* = 100)	RATHA (*n* = 100)	*p*-value
	Mean (SD)	Mean (SD)	
Hemoglobin (g/dl)			
Pre-operative	12.43 (1.4)	12.05 (1.5)	NS*
Post-op day 1	10 (1.3)	10.55 (1.4)	0.0072*
Post-op day 3	9.05 (1.3)	9.56 (1.3)	**0.0110** *
Largest drop in Hb (g/dL)	3.38 (1)	2.49 (0.6)	<0.001*
Blood volume estimation – Nadler method (mL)	4746.8 (530.8)	4304.6 (411)	<0.001*
Requirement of blood transfusion	*N* (%)	*N* (%)	
Yes	18	7	**0.0175†**
No	70	81	

* Student *t-*test;

† Chi-square test.

Bold values indicate statistical significance. NS stands for “Non-Significant”.

### Blood loss

**Figure 2:**
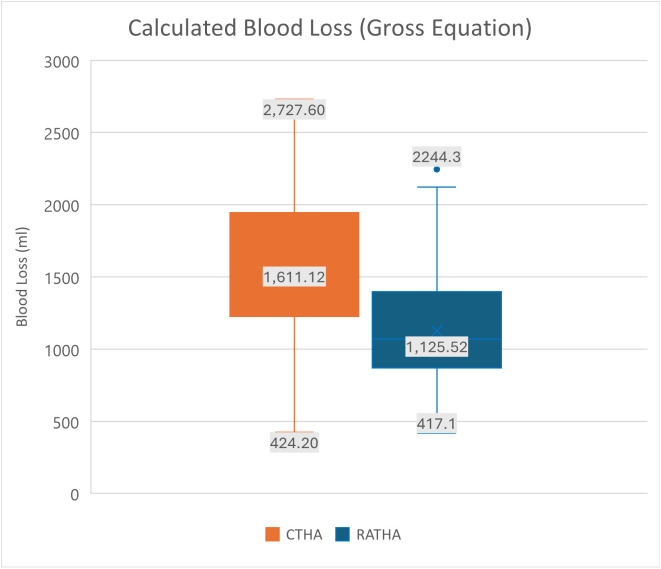
Mean blood loss (in mL) calculated using the Gross equation in both cohorts.

The mean blood volume was estimated using the Nadler method and the mean blood loss was estimated using the Gross equation. On the first post-operative day, CTHA group had a mean blood loss of 1153.9 mL (SD = 609.9); whereas the RATHA cohort had a significantly lower blood loss of 751.2 mL (SD = 375.9) (*p* < 0.0001). A similar trend was seen on the third post-operative day, where the CTHA group had a mean blood loss of 1611.12 mL (SD = 501.4); whereas the RATHA cohort had a significantly lower blood loss of 1125.52 mL (SD = 361.2) (*p* < 0.0001) (Table 3 and Figure 2). Relative blood loss was also found to be higher in the CTHA cohort (Day 1 – 24.2%, Day 3 – 34.1%) compared to the RATHA cohort (Day 1 – 17.05%, Day 3 – 25.68%) (Table 3).

**Table 3:** Estimated blood loss and relative blood loss percentage among CTHA and RATHA.

	CTHA (*n* = 88)		RATHA (*n* = 88)		*p*-value
	Blood loss estimation – Gross equation (mL)	Relative blood loss percentage	Blood loss estimation – Gross equation (mL)	Relative blood loss percentage	
Post-op day 1; Mean (SD)	1153.9 (609.9)	24.24 (12.3)	751.2 (375.9)	17.05 (8.4)	<0.001*
Post-op day 3; Mean (SD)	1611.1 (501.4)	34.11 (10.5)	1125.5 (361.2)	25.68 (7.6)	<0.001*

* Student *t-*test.

Bold values indicate statistical significance.

**Figure 3:**
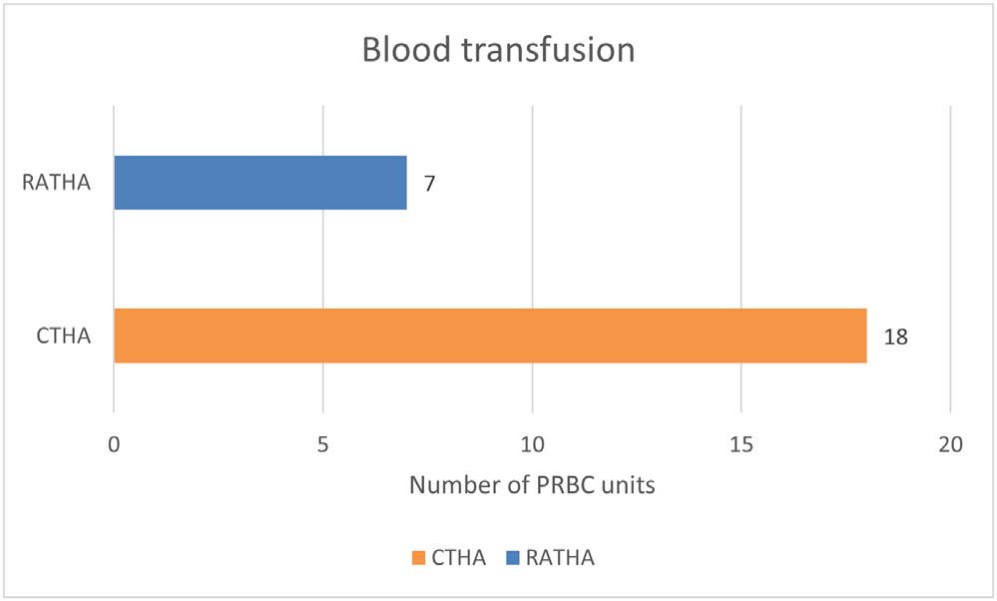
Number of units of packed red cell transfusion in both cohorts.

Transfusion rates were significantly higher in CTHA (*N* = 18, 20.4%) compared to RATHA (*N* = 7, 7.96%) (*p* = 0.0175) (Table 2 and Figure 3). There were no transfusion related adverse events reported in either group.

### Operative times

**Figure 4:**
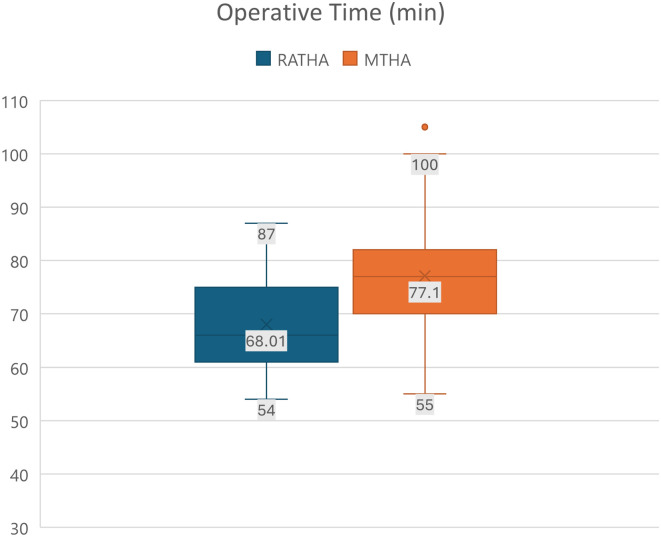
Mean operative times (skin-to-skin) time in both cohorts.

The mean operative times (skin-to-skin time) in the RATHA cohort was found to be 68.01 (SD = 8.7) min; which was significantly lower than the conventional cohort who had a mean operating time of 77.1 (SD = 10.5) min (*p* < 0.0001) (Figure 4).

### Length of stay

In the robotic cohort, all patients were discharged within the expected time of 3 days. However, in the MTHA cohort, 7 (14%) patients had to stay for an additional 2 days due to requirement of blood transfusions.

### Complications

None of the patients in either cohort developed any intra-operative or perioperative medical complications. One patient each in the robotic and conventional cohorts developed a transient partial sciatic nerve palsy with foot drop.

### Multivariate regression analysis

In the multivariate regression analysis (Table 4), cohort type, age, and BMI were significant predictors of blood loss. Specifically, the robotic cohort was associated with a substantial and statistically significant reduction in blood loss compared to the conventional cohort (coefficient = −6.43, *p* < 0.0001), indicating a clear advantage in minimizing blood loss with robotic assistance. Age had a minor but significant negative effect on blood loss (coefficient = −0.10, *p* = 0.0434). Conversely, BMI was positively associated with blood loss (coefficient = 0.78, *p* = 0.0018), suggesting higher blood loss in patients with greater BMI. In contrast, ASA grade (*p* = 0.0947) and pre-operative hemoglobin (*p* = 0.4849) were not significantly associated with blood loss, indicating that these factors had limited influence in this model.

**Table 4:** Multivariate regression analysis results.

Variable	Coefficient	*p*-value
Cohort (Robotic vs. Conventional)	−6.43	**0.0000**
Age	−0.10	**0.0434**
BMI	0.78	**0.0018**
ASA Grade	6.95	0.0947
Pre-op hemoglobin	0.33	0.4849

Bold values indicate statistical significance.

## Discussion

This prospective matched cohort-study showed a significant reduction in blood loss in patients undergoing RATHA compared to manual conventional THA. The mean drop in hemoglobin was significantly lower in the RATHA cohort. It was also observed that the maximal allowable blood loss as calculated using the Gross equation and the number blood transfusions were significantly lower in the RATHA cohort.

There is an increase in the adoption of robotic technology assistance during surgery across all the surgical sub-specialties [21, 22]. Studies have reported the benefits of robotics, including improved accuracy of component positioning, reduced risk of outliers and mal-alignment, reduced post-operative pain and improved patient outcomes [23, 24]. Despite these benefits, concerns remain about the technical complexity, learning curve, increased operative times, blood loss, complications, and overall costs to the patient and care providers [25–27].

Most literature pertaining to reduced blood loss with robotics are related to TKA with limited evidence supporting reduced blood loss after robotic THA. However, our findings corroborate some of the earlier studies on the use of robot-assisted technology in TKA wherein significant reductions in perioperative blood loss and blood transfusions have been reported [17, 28, 29].

Perioperative blood loss in THA poses a substantial risk to patient outcomes, with reported estimates of blood loss ranging between 1200 and 1700 mL, representing up to 20% of an individual’s total blood volume [30–32]. The management of blood loss is essential, necessitating a multimodal approach to blood management. Such strategies include achieving effective hemostasis, the administration of TXA, prophylaxis for DVT, and the employment of red cell salvage techniques to reduce the dependency on allogeneic transfusions.

Despite the availability of various formulas designed to assess surgical blood loss, the absence of a consensus on a gold standard remains a notable gap in the literature [33]. Accurate estimation of blood loss is crucial not only for minimizing or optimizing transfusion requirements to reduce patient morbidity but also for its potential to significantly lower the costs associated with healthcare provision. Enhancing the accuracy of blood loss measurement and improving blood management practices in THA are therefore imperative for improving patient care and promoting economic efficiency within the healthcare system. Cho et al. showed that the maximum post-operative drop in hemoglobin is seen on the third post-operative day, hence it is remains to vital to keep this in mind before planning blood transfusion and certifying patients for discharge [34]. This was in agreement with our study where the maximum drop in hemoglobin was seen on the third post-operative day and was found to be significantly lower in the RATHA cohort (CTHA – 3.38 vs. RATHA – 2.49, *p* < 0.0001).

Lee et al. showed a mean 2.5 g drop in Hb 48 h after THA which is consistent with our study findings [35]. Rodríguez-González et al. in a similar study on early experience with robotic assisted THA using MAKO reported a mean post-operative hemoglobin drop of 3.08 g/dL at 24 h which is larger than in our study [36].

Most studies have shown that pre-operative hemoglobin remains the single most important factor to determine blood transfusion post-surgery [37, 38]. A majority of our patients (72.7%, *N* = 128) had pre-operative hemoglobin levels below 13 g/dL. According to To et al., this Hb level carries a 45% risk of transfusion after THA [38]. Caldora et al. conducted a retrospective comparative study on 1537 patients who underwent either conventional or robotic uncemented total hip arthroplasty (THA). The study found that patients in the robotic cohort had a significantly lower blood transfusion rate, with only 2% requiring transfusion, compared to 11.2% in the conventional cohort (*p* < 0.001). The risk of transfusion was six times higher in the conventional THA group than in the robotic group. Even after adjusting for age and sex, there was still a 4.69-fold higher likelihood of transfusion with the conventional technique [39]. This finding is consistent with our study, which also found a statistically significant reduction in the need for blood transfusion in the robotic cohort. Similar high transfusions rates comparable to this study were reported in previous studies in the Indian population, reported by Vijay et al. [40] with a 16% transfusion rate, and Malhotra et al. [41] who reported transfusion rates of 24% and also other studies which have reported transfusion rates as high as 26% [37, 42].

The reason for reduction in blood loss in RATHA remains uncertain. It can be primarily attributed to the use of a single ream during the placement of the acetabular cup in RATHA. This approach enables the cup to be reamed and impacted with millimeter precision, thereby avoiding the need for unnecessary sequential reaming that is often required in the conventional technique.

Use of RATHA has also been shown to accelerate patient recovery often promoting early discharge from hospital thereby reducing length of stay in hospital [24, 39]. The use of robotic systems involves a learning curve that has been well documented. Heng et al. in their retrospective review reported that the robotic-assisted cohort had faster operating times than the manual cohort by 2 min (Robotic − 82.9 min vs. Conventional – 84.9 min), which is consistent with the findings of our study. This study also reported shorter length of stay with robotic-assisted THA compared to the conventional group. However, there were no significant differences in blood transfusion rates between the two groups [43]. A recent retrospective study found that the length of hospital stay is a significant risk factor in increasing the overall cost of primary THA [44].

None of the patients in this study had any medical or surgical complications with either technique and there was no incidence of DVT or pulmonary embolism with the aid of robotic technology for THA. Reducing the number of blood transfusions in patients undergoing THA can have several benefits, including shorter hospital stays which translates to direct cost reduction for patients. Although the initial investment cost of the Robotic system may be high, it can prove to be beneficial in the long run [39].

The main strengths of the study include a single-surgeon series of cases, with uniform peri-operative anesthetic, analgesic and rehabilitation protocols implemented across both cohorts. Patients also had similar gender distribution, comorbidities, ASA score, and pre-op indications. All patients received the same dose of TXA, and DVT prophylaxis regimen and received similar rehabilitation.

This study has some limitations. The main limitation of the study in this was a single-centre study with a relatively small sample size and larger multi-centre RCTs are needed to validate these findings. Second, considering the demographic included only Asian-Indian patients, with potential differences in baseline nutritional status, hematocrit and hemoglobin compared to other ethnic groups, the findings may not be generalizable. Third, it should be noted that the learning curve for RATHA was not considered in this study, as it was conducted three years after acquisition of robotics at our institute and all robotic operative times were recorded after placement of arrays which may take a few extra minutes. With experience and improved efficiency, we have shorter operative times with the aid of robotics. We attribute this to single reaming of the acetabulum and direct implantation of the final components before trialing. Finally, although a matched population was used for this analysis, high transfusion rates in the study maybe attributable to lack of pre-operative optimization of patients in the Indian setting with many patients having less than 13 g/dL of hemoglobin pre-operatively and lack of use of an extended TXA protocol.

## Conclusion

Robotic-assisted total hip arthroplasty was associated with reduced blood loss, a smaller drop in hemoglobin, and a lower requirement for blood transfusions. These advantages imply that the use of robotic technology has the potential to reduce patient morbidity, shorten hospital stays, and lessen the burden on the health system. However, it is crucial to conduct further research to verify its long-term effectiveness and safety in THA.

## Data Availability

Data and materials are available on request.
